# Crystal structure of *catena*-poly[[[aqua­lithium(I)]-μ-pyrimidine-2-carboxyl­ato-κ^4^ *N* ^1^,*O* ^2^:*N* ^3^,*O* ^2′^] hemihydrate]

**DOI:** 10.1107/S2056989014026735

**Published:** 2015-01-01

**Authors:** Wojciech Starosta, Janusz Leciejewicz

**Affiliations:** a Institute of Nuclear Chemistry & Technology, ul. Dorodna 16, 03-195 Warszawa, Poland

**Keywords:** crystal structure, one-dimensional coordination polymer, lithium compound, pyrimidine-2-carboxyl­ate, hydrogen bonding

## Abstract

The title compound, {[Li(C_5_H_3_N_2_O_2_)(H_2_O)]·0.5H_2_O}_*n*_, comprises four symmetry-independent Li(C_5_H_3_N_2_O_2_)(H_2_O) units which form mol­ecular ribbons running along the *c*-axis direction. Within each ribbon, the ligand mol­ecule, acting in a μ_2_-mode, bridges two adjacent Li^+^ cations using both of its *N*,*O*-bonding sites. The coordination environment of each of the four Li^+^ cations can be described alternatively as either slightly distorted trigonal–bipyramidal or slightly distorted square–pyramidal. The ribbons are inter­connected by a network of O—H⋯O hydrogen bonds.

## Abstract 2

In this one-dimensional coordination polymer, four symmetry-independent Li(C_5_H_3_N_2_O_2_)(H_2_O) units form mol­ecular ribbons running along the unit-cell *c*-axis direction. Within each ribbon, the ligand mol­ecule, acting in a μ_2_-mode, bridges two adjacent Li cations using both its *N*,*O*-bonding sites.

## Chemical context   

The pyrimidine-2-carboxyl­ato ligand exhibits rich versatility when applied to the synthesis of functional materials, resulting in structures with inter­esting structural and magnetic properties. Zeolite-type structures have been reported for Cd^II^ coordination polymers with this ligand (Sava *et al.*, 2008 ▸; Zhang *et al.*, 2008*a* ▸). A variety of polymeric mol­ecular patterns have been observed in the structures of a number of divalent metal complexes with the title ligand, for example: Mn^II^ (Rodríguez-Diéguez *et al.*, 2008 ▸; Zhang *et al.*, 2008*b* ▸); Fe^II^ and Co^II^ (Rodríguez-Diéguez *et al.*, 2007 ▸; Zhao & Liu, 2010 ▸); Ca^II^ (Zhang *et al.*, 2008*b* ▸); Cu^II^ (Suárez-Varela *et al.*, 2008 ▸). Polymeric mol­ecular patterns were also found in two Li^I^ structures with the pyrimidine-2-carboxyl­ato ligand (Starosta & Lecieje­wicz, 2011 ▸, 2012 ▸). Inter­esting hexa­nuclear, wheel-shaped nickel cationic complexes with the pyrimidine-2-carboxyl­ato ligand, encapsulating ClO_4_^−^ or BF_4_^−^ anions have been synthesized (Colacio *et al.*, 2009 ▸). Structures built of monomeric mol­ecules have been also reported in an Ag^I^ complex by Kokunov & Gorbunova (2007 ▸) and in a Cu^II^ complex by Suárez-Varela *et al.* (2008 ▸) and Zhang *et al.* (2008*c* ▸).
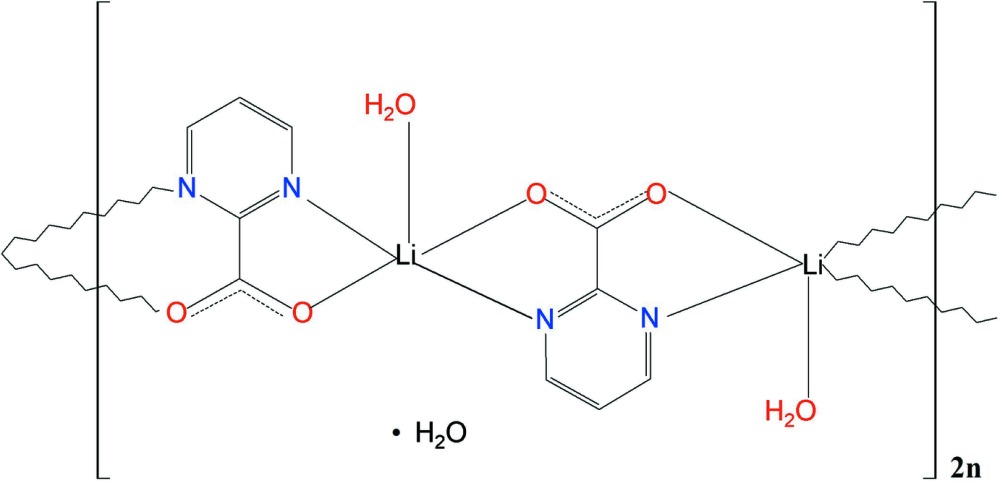


In the course of our studies of coordination modes of lithium complexes with diazine carboxyl­ates, a third lithium complex with the title ligand has recently been synthesized.

## Structural commentary   

**Figure 1:**
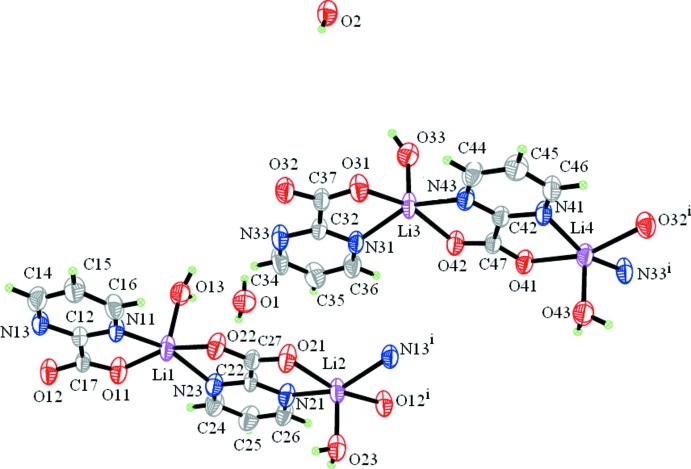
Fragments of two mol­ecular ribbons in the structure of the title compound, showing the atom labels and 50% probability displacement ellipsoids for the non-H atoms. [Symmetry codes: (i) *x*, *y*, *z* + 1; (ii) *x*, *y*, *z* − 1.]

**Figure 2:**
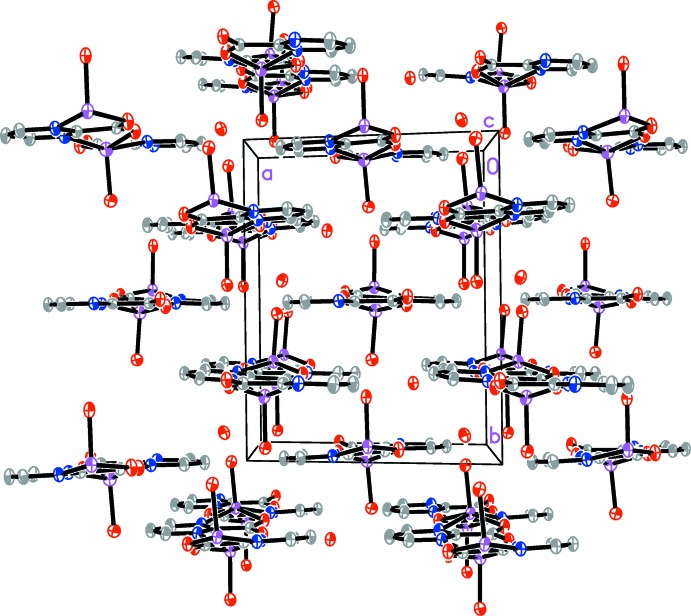
The packing of mol­ecular ribbons in the structure of the title compound as viewed down the ribbon direction (the crystallographic *c* axis). For clarity, H atoms are not shown.

A mol­ecular assembly consisting of an aqua-coordinated Li^I^ cation and a bonded pyrimidine-2-carboxyl­ate (C_5_H_3_N_2_O_2_) ligand constitutes the structural unit of the title polymeric compound, {[Li(C_5_H_3_N_2_O_2_)(H_2_O)]·0.5H_2_O}_*n*_. There are four such assemblies in the asymmetric unit. Linked into pairs, they form mol­ecular ribbons in which the (C_5_H_3_N_2_O_2_) ligand bridges adjacent Li^I^ cations using both its *N*,*O* bonding sites (μ_2_-bridging mode) (Fig. 1 ▸). The ribbons propagate in the *c*-axis direction (Fig. 2 ▸).

All four Li^I^ cations show a penta-coordination mode which can be described by two alternative geometries: either trigonal–bipyramidal or square–pyramidal, both slightly deformed. For example, in the case of the Li1 cation, the equatorial plane of a trigonal bipyramid consists of atoms O13, N11 and N23 with Li1 0.0712 (5) Å out of this plane; atoms O11 and O22 are at the apices. On the other hand, the base of the square pyramid is formed by the O11, O22, N11 and N23 atoms [r.m.s. 0.0069 (1) Å], O13 is at the apex; the Li1 cation is 0.3989 (8) Å out of the base. A similar description can be made for the remaining three independent LiO_3_N_2_ groups. The Li—O and Li—N bond lengths (Table 1 ▸) fall in the range commonly observed in other Li complexes with the title ligand (Starosta & Leciejewicz, 2011 ▸, 2012 ▸). The pyrimidine rings of all four ligand mol­ecules are almost planar, with r.m.s. deviations ranging from 0.0024 (1) (ligand 4) to 0.0094 (1) Å (ligand 1). The carboxyl­ate groups make dihedral angles with hetero-rings in the range from 2.8 (1) (ligand 2) to 7.6 (1)° (ligand 1).

## Supra­molecular features   

The ribbons inter­act *via* a network of hydrogen bonds (Table 2 ▸). Water mol­ecules of solvation act as donors, while the carboxyl­ate O atoms from adjacent ribbons act as acceptors. Hydrogen bonds between coordinating water mol­ecules as donors and carboxyl­ate O atoms belonging to adjacent ribbons as acceptors are also observed.

## Related complexes   

The title compound is the third Li complex with the pyrimidine-2-carboxyl­ate ligand reported so far. In one of these complexes (Starosta & Leciejewicz, 2011 ▸), mol­ecular ribbons composed of Li cations bridged by the bidentate carboxyl­ate groups and bridged by bidentate nitrate anions form mol­ecular layers. An inter­esting feature is the absence of any *N*,*O* chelating bonding to the metal ion. The structural motif in the remaining complex (Starosta & Leciejewicz, 2012 ▸) consists of a mol­ecular chain similar to that in the title compound. In this structure, the chains are bridged by pairs of aqua-coordinated Li ions inter-connected by an aqua O atom. The tetra­hedral coordination of each of these Li cations is completed by two carboxyl­ate O atoms acting in a bidentate mode and donated by the ligands belonging to adjacent chains. The charge of the resulting cationic ribbon is compensated by the inter­spersed chloride anions.

## Synthesis and crystallization   

50 ml of an aqueous solution containing 1 mmol of pyrimidine-2-carbo­nitrile and 5 mmol of LiOH was boiled under reflux for 20 h with constant stirring. After cooling to room temperature, the solution was filtered and titrated with 0.1 *N* acetic acid until the pH reached *ca* 6.5, then stirred at 320 K for 3 h and left to evaporate slowly at room temperature. The residue was redissolved in a 1:1 ethanol–water mixture and left to crystallize at room temperature. After a few days, block-shaped single crystal of the title compound were extracted, washed with cold methanol and dried in the air.

## Refinement   

Crystal data, data collection and structure refinement details are summarized in Table 3 ▸. H atoms bonded to pyridine-ring C atoms were placed at calculated positions with C—H = 0.93 Å and treated as riding on the parent atoms with *U*_iso_(H) = 1.2*U*_eq_(C). The H atoms of water mol­ecules were found from the Fourier map and refined isotropically.

## Supplementary Material

Crystal structure: contains datablock(s) I, global. DOI: 10.1107/S2056989014026735/bg2542sup1.cif

Structure factors: contains datablock(s) I. DOI: 10.1107/S2056989014026735/bg2542Isup2.hkl

CCDC reference: 1037774

Additional supporting information:  crystallographic information; 3D view; checkCIF report

## Figures and Tables

**Table 1:** Selected bond lengths ()

Li1O13	2.012(14)	Li3O33	2.002(13)
Li1O11	2.030(10)	Li3O31	2.107(10)
Li1N23	2.111(11)	Li3O42	2.103(10)
Li1N11	2.121(11)	Li3N43	2.154(9)
Li1O22	2.154(10)	Li3N31	2.164(9)
Li2O23	1.996(12)	Li4O43	2.010(12)
Li2O12	2.077(10)	Li4O32	2.092(9)
Li2O21^i^	2.094(10)	Li4N41^i^	2.107(10)
Li2N13	2.138(9)	Li4N33	2.120(10)
Li2N21^i^	2.180(9)	Li4O41^i^	2.126(9)

Symmetry code: (i) 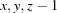.

**Table 2:** Hydrogen-bond geometry (, )

*D*H*A*	*D*H	H*A*	*D* *A*	*D*H*A*
O1H11O31	0.86(2)	1.99(3)	2.814(7)	159(8)
O1H12O22^ii^	0.86(2)	2.06(2)	2.897(8)	164(6)
O2H21O32^ii^	0.86(2)	2.04(3)	2.849(7)	155(7)
O2H22O21^iii^	0.86(2)	1.90(2)	2.755(7)	174(8)
O13H131O41^i^	0.86(1)	2.13(3)	2.898(6)	149(4)
O13H132O1^iv^	0.86(2)	2.02(3)	2.867(6)	165(7)
O23H232O13^v^	0.86(2)	2.01(3)	2.807(6)	154(5)
O33H331O12^vi^	0.86(2)	1.93(2)	2.777(7)	169(6)
O33H332O43^ii^	0.85(2)	2.31(3)	3.106(6)	154(6)
O43H431O22	0.86(2)	2.03(2)	2.879(6)	170(7)
O43H432O2^vii^	0.86(1)	2.00(4)	2.773(6)	148(5)
O23H231O42^viii^	0.86(1)	1.86(2)	2.715(6)	177(5)

Symmetry codes: (i) 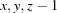; (ii) 
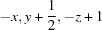
; (iii) 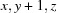; (iv) 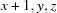; (v) 
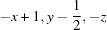
; (vi) 
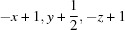
; (vii) 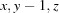; (viii) 
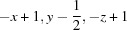
.

**Table 3:** Experimental details

Crystal data	
Chemical formula	[Li(C_5_H_3_N_2_O_2_)(H_2_O)]0.5H_2_O
*M*_r_	157.06
Crystal system, space group	Monoclinic, *P*2_1_
Temperature (K)	293
*a*, *b*, *c* ()	10.4965(5), 12.8118(6), 10.8810(4)
()	107.771(5)
*V* (^3^)	1393.45(11)
*Z*	8
Radiation type	Cu *K*
(mm^1^)	1.07
Crystal size (mm)	0.17 0.08 0.05

Data collection	
Diffractometer	Agilent CCD Xcalibur Ruby
Absorption correction	Analytical [*CrysAlis PRO* (Agilent, 2014 ▸), based on expressions derived by Clark Reid (1995 ▸)]
*T*_min_, *T*_max_	0.894, 0.952
No. of measured, independent and observed [*I* > 2(*I*)] reflections	10782, 5237, 3736
*R*_int_	0.056
(sin /)_max_ (^1^)	0.614

Refinement	
*R*[*F* ^2^ > 2(*F* ^2^)], *wR*(*F* ^2^), *S*	0.061, 0.177, 0.98
No. of reflections	5237
No. of parameters	451
No. of restraints	20
H-atom treatment	H atoms treated by a mixture of independent and constrained refinement
_max_, _min_ (e ^3^)	0.35, 0.23

Computer programs: *CrysAlis PRO* (Agilent, 2014 ▸), *SHELXS2014* and *SHELXL2014* (Sheldrick, 2008 ▸).

## supplementary crystallographic information

### Crystal data

**Table:**

[Li(C_5_H_3_N_2_O_2_)(H_2_O)]·0.5H_2_O	*F*(000) = 648
*M**_r_* = 157.06	*D*_x_ = 1.497 Mg m^−^^3^
Monoclinic, *P*2_1_	Cu *K*α radiation, λ = 1.54184 Å
*a* = 10.4965 (5) Å	Cell parameters from 2423 reflections
*b* = 12.8118 (6) Å	θ = 4.4–70.6°
*c* = 10.8810 (4) Å	µ = 1.07 mm^−^^1^
β = 107.771 (5)°	*T* = 293 K
*V* = 1393.45 (11) Å^3^	Block, colourless
*Z* = 8	0.17 × 0.08 × 0.05 mm

### Data collection

**Table:**

Agilent CCD Xcalibur Ruby diffractometer	5237 independent reflections
Radiation source: Enhance (Cu) X-ray Source	3736 reflections with *I* > 2σ(*I*)
Detector resolution: 10.4922 pixels mm^-1^	*R*_int_ = 0.056
ω scans	θ_max_ = 71.2°, θ_min_ = 4.3°
Absorption correction: analytical [*CrysAlis PRO* (Agilent, 2014), based on expressions derived by Clark & Reid (1995)]	*h* = −11→12
*T*_min_ = 0.894, *T*_max_ = 0.952	*k* = −15→15
10782 measured reflections	*l* = −13→12

### Refinement

**Table:**

Refinement on *F*^2^	20 restraints
Least-squares matrix: full	Hydrogen site location: mixed
*R*[*F*^2^ > 2σ(*F*^2^)] = 0.061	H atoms treated by a mixture of independent and constrained refinement
*wR*(*F*^2^) = 0.177	*w* = 1/[σ^2^(*F*_o_^2^) + (0.1*P*)^2^] where *P* = (*F*_o_^2^ + 2*F*_c_^2^)/3
*S* = 0.98	(Δ/σ)_max_ = 0.004
5237 reflections	Δρ_max_ = 0.35 e Å^−^^3^
451 parameters	Δρ_min_ = −0.23 e Å^−^^3^

### Special details

**Table:**

Experimental. Absorption correction: Agilent (2014). Clark & Reid (1995). Analytical numeric absorption correction using a multifaceted crystal model based on expressions derived by R.C. Clark & J.S. Reid. Empirical absorption correction using spherical harmonics, implemented in SCALE3 ABSPACK scaling algorithm.
Geometry. All esds (except the esd in the dihedral angle between two l.s. planes) are estimated using the full covariance matrix. The cell esds are taken into account individually in the estimation of esds in distances, angles and torsion angles; correlations between esds in cell parameters are only used when they are defined by crystal symmetry. An approximate (isotropic) treatment of cell esds is used for estimating esds involving l.s. planes.
Refinement. Refinement of *F*^2^ against ALL reflections. The weighted *R*-factor *wR* and goodness of fit *S* are based on *F*^2^, conventional *R*-factors *R* are based on *F*, with *F* set to zero for negative *F*^2^. The threshold expression of *F*^2^ > σ(*F*^2^) is used only for calculating *R*-factors(gt) *etc*. and is not relevant to the choice of reflections for refinement. *R*-factors based on *F*^2^ are statistically about twice as large as those based on *F*, and *R*- factors based on ALL data will be even larger.

### Fractional atomic coordinates and isotropic or equivalent isotropic displacement parameters (Å^2^)

**Table:**

	*x*	*y*	*z*	*U*_iso_*/*U*_eq_	
Li1	0.5210 (11)	0.0381 (10)	0.3508 (8)	0.054 (3)	
Li2	0.5170 (10)	−0.0392 (9)	−0.1441 (8)	0.046 (2)	
Li3	0.0814 (10)	0.2948 (9)	0.8783 (8)	0.047 (2)	
Li4	0.0921 (9)	0.2019 (9)	0.3830 (7)	0.043 (2)	
C12	0.4379 (6)	0.0069 (5)	0.0797 (5)	0.0384 (12)	
C14	0.2428 (6)	0.0067 (6)	−0.0846 (6)	0.0529 (15)	
H14	0.1963	0.0069	−0.1723	0.064*	
C15	0.1725 (7)	0.0078 (6)	0.0028 (6)	0.0530 (16)	
H15	0.0795	0.0100	−0.0239	0.064*	
C16	0.2446 (6)	0.0055 (6)	0.1314 (6)	0.0489 (15)	
H16	0.1994	0.0029	0.1927	0.059*	
C17	0.5892 (6)	0.0064 (5)	0.1248 (5)	0.0378 (13)	
C22	0.5980 (6)	−0.0002 (5)	0.6247 (5)	0.0370 (12)	
C24	0.7913 (7)	0.0111 (6)	0.5742 (6)	0.0537 (16)	
H24	0.8369	0.0147	0.5132	0.064*	
C25	0.8632 (7)	0.0101 (6)	0.7026 (6)	0.0569 (16)	
H25	0.9561	0.0141	0.7299	0.068*	
C26	0.7921 (6)	0.0029 (6)	0.7891 (5)	0.0503 (15)	
H26	0.8387	0.0013	0.8768	0.060*	
C27	0.4461 (6)	−0.0097 (5)	0.5817 (5)	0.0392 (14)	
C32	0.1661 (6)	0.2465 (4)	0.6557 (5)	0.0380 (12)	
C34	0.3599 (7)	0.2476 (7)	0.6072 (6)	0.0625 (19)	
H34	0.4067	0.2477	0.5471	0.075*	
C35	0.4314 (7)	0.2511 (7)	0.7353 (6)	0.0598 (18)	
H35	0.5244	0.2529	0.7632	0.072*	
C36	0.3584 (6)	0.2519 (6)	0.8201 (5)	0.0530 (15)	
H36	0.4038	0.2534	0.9081	0.064*	
C37	0.0137 (7)	0.2451 (5)	0.6084 (5)	0.0410 (14)	
C42	0.0088 (6)	0.2435 (5)	1.1091 (5)	0.0365 (13)	
C44	−0.1857 (6)	0.2422 (5)	0.9473 (5)	0.0472 (14)	
H44	−0.2328	0.2457	0.8598	0.057*	
C45	−0.2559 (7)	0.2292 (6)	1.0347 (7)	0.0531 (16)	
H45	−0.3487	0.2239	1.0082	0.064*	
C46	−0.1812 (7)	0.2244 (6)	1.1641 (6)	0.0484 (16)	
H46	−0.2253	0.2164	1.2259	0.058*	
C47	0.1617 (6)	0.2510 (5)	1.1531 (5)	0.0380 (13)	
O1	−0.3233 (5)	0.2775 (4)	0.5971 (5)	0.0595 (11)	
H11	−0.240 (3)	0.263 (5)	0.608 (8)	0.071*	
H12	−0.327 (6)	0.3439 (15)	0.586 (8)	0.071*	
O2	0.1225 (5)	0.9356 (4)	0.6054 (4)	0.0590 (12)	
H21	0.120 (6)	0.8692 (15)	0.593 (8)	0.071*	
H22	0.205 (3)	0.952 (5)	0.619 (8)	0.071*	
O11	0.6455 (4)	0.0173 (4)	0.2421 (4)	0.0535 (12)	
O12	0.6448 (4)	−0.0058 (4)	0.0388 (4)	0.0508 (11)	
O13	0.5044 (5)	0.1941 (4)	0.3601 (4)	0.0530 (11)	
H131	0.431 (3)	0.225 (5)	0.3553 (17)	0.064*	
H132	0.563 (4)	0.226 (5)	0.422 (5)	0.064*	
O21	0.3915 (5)	−0.0202 (4)	0.6665 (4)	0.0539 (13)	
O22	0.3905 (4)	−0.0051 (4)	0.4616 (4)	0.0513 (11)	
O23	0.5207 (4)	−0.1946 (4)	−0.1316 (4)	0.0498 (11)	
H231	0.6051 (18)	−0.207 (6)	−0.113 (5)	0.060*	
H232	0.487 (5)	−0.225 (5)	−0.205 (4)	0.060*	
O31	−0.0433 (4)	0.2566 (4)	0.6921 (4)	0.0510 (11)	
O32	−0.0389 (4)	0.2317 (4)	0.4901 (4)	0.0514 (11)	
O33	0.0834 (5)	0.4508 (4)	0.8678 (4)	0.0516 (12)	
H331	0.166 (2)	0.471 (5)	0.889 (6)	0.062*	
H332	0.043 (5)	0.495 (4)	0.811 (5)	0.062*	
O41	0.2199 (4)	0.2372 (4)	1.2700 (3)	0.0458 (11)	
O42	0.2119 (4)	0.2694 (4)	1.0654 (4)	0.0468 (10)	
O43	0.1100 (4)	0.0457 (4)	0.3826 (4)	0.0518 (11)	
H431	0.1952 (16)	0.038 (6)	0.410 (6)	0.062*	
H432	0.082 (5)	0.007 (5)	0.434 (5)	0.062*	
N11	0.3768 (5)	0.0070 (5)	0.1701 (4)	0.0422 (12)	
N13	0.3764 (5)	0.0054 (4)	−0.0470 (4)	0.0439 (11)	
N21	0.6594 (5)	−0.0019 (4)	0.7526 (4)	0.0445 (11)	
N23	0.6582 (5)	0.0071 (5)	0.5341 (4)	0.0476 (12)	
N31	0.2263 (5)	0.2508 (4)	0.7826 (4)	0.0429 (11)	
N33	0.2279 (5)	0.2440 (5)	0.5641 (5)	0.0505 (13)	
N41	−0.0494 (5)	0.2310 (4)	1.2018 (4)	0.0429 (12)	
N43	−0.0532 (5)	0.2498 (4)	0.9838 (4)	0.0423 (11)	

### Atomic displacement parameters (Å^2^)

**Table:**

	*U*^11^	*U*^22^	*U*^33^	*U*^12^	*U*^13^	*U*^23^
Li1	0.060 (7)	0.078 (8)	0.027 (5)	0.000 (5)	0.020 (5)	0.001 (4)
Li2	0.046 (6)	0.069 (7)	0.029 (4)	0.001 (4)	0.019 (4)	−0.002 (4)
Li3	0.046 (6)	0.075 (7)	0.025 (4)	−0.005 (5)	0.017 (4)	−0.005 (4)
Li4	0.040 (5)	0.064 (6)	0.027 (4)	0.003 (4)	0.010 (4)	0.003 (4)
C12	0.046 (3)	0.042 (3)	0.028 (2)	0.003 (3)	0.013 (2)	−0.002 (2)
C14	0.045 (3)	0.076 (4)	0.033 (3)	0.008 (3)	0.005 (2)	−0.004 (3)
C15	0.036 (3)	0.080 (5)	0.041 (3)	0.003 (3)	0.009 (3)	−0.006 (3)
C16	0.043 (3)	0.069 (4)	0.041 (3)	−0.001 (3)	0.022 (3)	−0.006 (3)
C17	0.044 (3)	0.047 (3)	0.025 (2)	0.001 (3)	0.014 (2)	0.001 (2)
C22	0.045 (3)	0.046 (3)	0.022 (2)	0.000 (3)	0.014 (2)	0.000 (2)
C24	0.041 (3)	0.081 (4)	0.046 (3)	0.002 (3)	0.023 (3)	0.009 (3)
C25	0.036 (3)	0.087 (5)	0.044 (3)	0.000 (3)	0.007 (3)	0.008 (3)
C26	0.044 (3)	0.072 (4)	0.028 (3)	−0.006 (3)	0.002 (2)	0.002 (3)
C27	0.040 (3)	0.053 (4)	0.024 (3)	0.003 (3)	0.009 (2)	0.000 (2)
C32	0.042 (3)	0.047 (3)	0.025 (2)	−0.002 (2)	0.011 (2)	0.000 (2)
C34	0.048 (4)	0.106 (6)	0.042 (3)	−0.002 (4)	0.025 (3)	−0.010 (3)
C35	0.040 (3)	0.090 (5)	0.047 (3)	0.000 (3)	0.009 (3)	−0.014 (3)
C36	0.046 (3)	0.080 (4)	0.031 (2)	0.002 (3)	0.007 (2)	−0.007 (3)
C37	0.044 (3)	0.054 (4)	0.026 (3)	0.000 (3)	0.013 (2)	−0.003 (2)
C42	0.038 (3)	0.045 (3)	0.027 (2)	0.000 (2)	0.010 (2)	0.000 (2)
C44	0.041 (3)	0.065 (4)	0.031 (3)	−0.002 (3)	0.005 (2)	0.002 (3)
C45	0.035 (3)	0.074 (5)	0.049 (3)	−0.001 (3)	0.012 (3)	0.004 (3)
C46	0.043 (4)	0.072 (4)	0.033 (3)	−0.001 (3)	0.015 (3)	0.003 (3)
C47	0.040 (3)	0.048 (3)	0.027 (2)	0.001 (2)	0.011 (2)	0.000 (2)
O1	0.042 (2)	0.076 (3)	0.058 (3)	0.003 (2)	0.012 (2)	0.000 (2)
O2	0.051 (3)	0.080 (3)	0.047 (2)	−0.009 (2)	0.017 (2)	−0.002 (2)
O11	0.044 (2)	0.085 (3)	0.0309 (19)	−0.003 (2)	0.0103 (18)	−0.006 (2)
O12	0.040 (2)	0.081 (3)	0.0329 (19)	−0.001 (2)	0.0146 (17)	−0.005 (2)
O13	0.055 (3)	0.063 (3)	0.040 (2)	0.002 (2)	0.014 (2)	−0.0012 (18)
O21	0.040 (2)	0.092 (4)	0.031 (2)	−0.005 (2)	0.0123 (18)	0.004 (2)
O22	0.043 (2)	0.080 (3)	0.0290 (19)	−0.004 (2)	0.0082 (17)	0.0042 (19)
O23	0.037 (2)	0.081 (3)	0.0319 (19)	0.001 (2)	0.0114 (18)	−0.0022 (19)
O31	0.040 (2)	0.080 (3)	0.0360 (19)	−0.006 (2)	0.0154 (18)	−0.006 (2)
O32	0.041 (2)	0.081 (3)	0.0313 (19)	0.003 (2)	0.0094 (17)	−0.0048 (19)
O33	0.047 (3)	0.063 (3)	0.044 (2)	−0.001 (2)	0.013 (2)	0.0030 (19)
O41	0.038 (2)	0.071 (3)	0.0282 (19)	0.001 (2)	0.0091 (17)	0.0032 (18)
O42	0.038 (2)	0.073 (3)	0.0309 (17)	−0.001 (2)	0.0125 (16)	0.0052 (18)
O43	0.051 (2)	0.066 (3)	0.040 (2)	0.004 (2)	0.017 (2)	0.0062 (18)
N11	0.043 (3)	0.061 (3)	0.024 (2)	−0.004 (2)	0.012 (2)	−0.004 (2)
N13	0.044 (3)	0.061 (3)	0.027 (2)	0.002 (2)	0.011 (2)	−0.003 (2)
N21	0.040 (3)	0.065 (3)	0.027 (2)	−0.004 (2)	0.0085 (19)	0.000 (2)
N23	0.047 (3)	0.065 (3)	0.032 (2)	0.003 (3)	0.013 (2)	0.002 (2)
N31	0.043 (3)	0.063 (3)	0.023 (2)	0.004 (2)	0.0103 (19)	−0.001 (2)
N33	0.046 (3)	0.079 (4)	0.031 (2)	0.000 (3)	0.017 (2)	−0.004 (2)
N41	0.038 (3)	0.066 (3)	0.027 (2)	0.000 (2)	0.014 (2)	0.003 (2)
N43	0.041 (2)	0.058 (3)	0.028 (2)	0.002 (2)	0.011 (2)	0.002 (2)

### Geometric parameters (Å, º)

**Table:**

Li1—O13	2.012 (14)	C27—O21	1.233 (7)
Li1—O11	2.030 (10)	C27—O22	1.260 (7)
Li1—N23	2.111 (11)	C32—N31	1.333 (7)
Li1—N11	2.121 (11)	C32—N33	1.345 (7)
Li1—O22	2.154 (10)	C32—C37	1.524 (9)
Li2—O23	1.996 (12)	C34—N33	1.321 (9)
Li2—O12	2.077 (10)	C34—C35	1.368 (9)
Li2—O21^i^	2.094 (10)	C34—H34	0.9300
Li2—N13	2.138 (9)	C35—C36	1.367 (9)
Li2—N21^i^	2.180 (9)	C35—H35	0.9300
Li3—O33	2.002 (13)	C36—N31	1.321 (8)
Li3—O31	2.107 (10)	C36—H36	0.9300
Li3—O42	2.103 (10)	C37—O31	1.242 (8)
Li3—N43	2.154 (9)	C37—O32	1.248 (7)
Li3—N31	2.164 (9)	C42—N43	1.322 (7)
Li4—O43	2.010 (12)	C42—N41	1.339 (8)
Li4—O32	2.092 (9)	C42—C47	1.531 (8)
Li4—N41^i^	2.107 (10)	C44—N43	1.328 (8)
Li4—N33	2.120 (10)	C44—C45	1.379 (9)
Li4—O41^i^	2.126 (9)	C44—H44	0.9300
C12—N11	1.327 (8)	C45—C46	1.389 (9)
C12—N13	1.332 (7)	C45—H45	0.9300
C12—C17	1.512 (8)	C46—N41	1.321 (8)
C14—N13	1.336 (8)	C46—H46	0.9300
C14—C15	1.370 (9)	C47—O42	1.246 (7)
C14—H14	0.9300	C47—O41	1.244 (7)
C15—C16	1.372 (9)	O1—H11	0.861 (15)
C15—H15	0.9300	O1—H12	0.859 (15)
C16—N11	1.322 (8)	O2—H21	0.861 (15)
C16—H16	0.9300	O2—H22	0.857 (15)
C17—O11	1.240 (7)	O13—H131	0.857 (14)
C17—O12	1.255 (7)	O13—H132	0.864 (15)
C22—N23	1.327 (7)	O21—Li2^ii^	2.094 (10)
C22—N21	1.343 (7)	O23—H231	0.861 (14)
C22—C27	1.523 (8)	O23—H232	0.857 (15)
C24—N23	1.332 (8)	O33—H331	0.863 (15)
C24—C25	1.371 (9)	O33—H332	0.854 (15)
C24—H24	0.9300	O41—Li4^ii^	2.126 (9)
C25—C26	1.371 (9)	O43—H431	0.858 (15)
C25—H25	0.9300	O43—H432	0.862 (14)
C26—N21	1.328 (8)	N21—Li2^ii^	2.180 (9)
C26—H26	0.9300	N41—Li4^ii^	2.107 (10)
			
O13—Li1—O11	103.9 (5)	N33—C34—C35	123.6 (5)
O13—Li1—N23	100.6 (5)	N33—C34—H34	118.2
O11—Li1—N23	98.7 (5)	C35—C34—H34	118.2
O13—Li1—N11	100.7 (5)	C34—C35—C36	116.2 (6)
O11—Li1—N11	80.8 (4)	C34—C35—H35	121.9
N23—Li1—N11	158.2 (7)	C36—C35—H35	121.9
O13—Li1—O22	98.5 (5)	N31—C36—C35	122.9 (5)
O11—Li1—O22	157.6 (7)	N31—C36—H36	118.6
N23—Li1—O22	77.9 (3)	C35—C36—H36	118.6
N11—Li1—O22	94.3 (5)	O31—C37—O32	127.7 (6)
O23—Li2—O12	98.4 (5)	O31—C37—C32	116.3 (5)
O23—Li2—O21^i^	100.1 (5)	O32—C37—C32	116.0 (5)
O12—Li2—O21^i^	161.4 (7)	N43—C42—N41	126.2 (6)
O23—Li2—N13	103.6 (5)	N43—C42—C47	117.3 (5)
O12—Li2—N13	79.2 (3)	N41—C42—C47	116.6 (5)
O21^i^—Li2—N13	97.7 (4)	N43—C44—C45	122.2 (5)
O23—Li2—N21^i^	104.6 (5)	N43—C44—H44	118.9
O12—Li2—N21^i^	96.0 (4)	C45—C44—H44	118.9
O21^i^—Li2—N21^i^	78.0 (3)	C44—C45—C46	116.8 (6)
N13—Li2—N21^i^	151.8 (6)	C44—C45—H45	121.6
O33—Li3—O31	101.0 (5)	C46—C45—H45	121.6
O33—Li3—O42	101.2 (5)	N41—C46—C45	121.7 (6)
O31—Li3—O42	157.6 (7)	N41—C46—H46	119.1
O33—Li3—N43	108.5 (5)	C45—C46—H46	119.1
O31—Li3—N43	97.7 (4)	O42—C47—O41	128.3 (6)
O42—Li3—N43	77.4 (3)	O42—C47—C42	114.8 (5)
O33—Li3—N31	102.2 (5)	O41—C47—C42	116.8 (5)
O31—Li3—N31	78.4 (3)	H11—O1—H12	104 (2)
O42—Li3—N31	94.6 (4)	H21—O2—H22	104 (2)
N43—Li3—N31	149.2 (6)	C17—O11—Li1	115.2 (5)
O43—Li4—O32	105.1 (5)	C17—O12—Li2	115.4 (5)
O43—Li4—N41^i^	102.4 (5)	Li1—O13—H131	123 (4)
O32—Li4—N41^i^	95.4 (4)	Li1—O13—H132	117 (4)
O43—Li4—N33	102.6 (5)	H131—O13—H132	104 (2)
O32—Li4—N33	78.8 (3)	C27—O21—Li2^ii^	116.9 (5)
N41^i^—Li4—N33	155.0 (7)	C27—O22—Li1	114.9 (5)
O43—Li4—O41^i^	97.7 (4)	Li2—O23—H231	101 (5)
O32—Li4—O41^i^	157.1 (6)	Li2—O23—H232	113 (5)
N41^i^—Li4—O41^i^	79.1 (3)	H231—O23—H232	104 (2)
N33—Li4—O41^i^	96.9 (4)	C37—O31—Li3	115.8 (5)
N11—C12—N13	125.1 (6)	C37—O32—Li4	116.1 (5)
N11—C12—C17	117.2 (5)	Li3—O33—H331	108 (4)
N13—C12—C17	117.7 (5)	Li3—O33—H332	134 (5)
N13—C14—C15	121.7 (5)	H331—O33—H332	104 (2)
N13—C14—H14	119.1	C47—O41—Li4^ii^	115.0 (5)
C15—C14—H14	119.1	C47—O42—Li3	117.9 (4)
C14—C15—C16	117.4 (6)	Li4—O43—H431	102 (5)
C14—C15—H15	121.3	Li4—O43—H432	121 (5)
C16—C15—H15	121.3	H431—O43—H432	103 (2)
N11—C16—C15	121.6 (5)	C16—N11—C12	117.5 (5)
N11—C16—H16	119.2	C16—N11—Li1	132.9 (5)
C15—C16—H16	119.2	C12—N11—Li1	108.5 (5)
O11—C17—O12	126.7 (6)	C12—N13—C14	116.6 (5)
O11—C17—C12	117.2 (5)	C12—N13—Li2	109.0 (5)
O12—C17—C12	116.1 (5)	C14—N13—Li2	132.1 (5)
N23—C22—N21	125.7 (5)	C26—N21—C22	115.9 (5)
N23—C22—C27	117.9 (4)	C26—N21—Li2^ii^	132.6 (4)
N21—C22—C27	116.4 (4)	C22—N21—Li2^ii^	110.5 (5)
N23—C24—C25	122.0 (5)	C24—N23—C22	116.7 (5)
N23—C24—H24	119.0	C24—N23—Li1	130.3 (5)
C25—C24—H24	119.0	C22—N23—Li1	111.9 (5)
C26—C25—C24	117.0 (6)	C36—N31—C32	116.2 (5)
C26—C25—H25	121.5	C36—N31—Li3	132.4 (4)
C24—C25—H25	121.5	C32—N31—Li3	109.2 (4)
N21—C26—C25	122.6 (5)	C34—N33—C32	115.3 (5)
N21—C26—H26	118.7	C34—N33—Li4	132.3 (5)
C25—C26—H26	118.7	C32—N33—Li4	110.9 (4)
O21—C27—O22	127.4 (6)	C46—N41—C42	116.7 (5)
O21—C27—C22	117.4 (5)	C46—N41—Li4^ii^	130.7 (5)
O22—C27—C22	115.2 (5)	C42—N41—Li4^ii^	111.9 (5)
N31—C32—N33	125.8 (5)	C42—N43—C44	116.5 (5)
N31—C32—C37	117.9 (5)	C42—N43—Li3	111.7 (5)
N33—C32—C37	116.3 (5)	C44—N43—Li3	131.1 (4)

Symmetry codes: (i) *x*, *y*, *z*−1; (ii) *x*, *y*, *z*+1.

### Hydrogen-bond geometry (Å, º)

**Table:**

*D*—H···*A*	*D*—H	H···*A*	*D*···*A*	*D*—H···*A*
O1—H11···O31	0.86 (2)	1.99 (3)	2.814 (7)	159 (8)
O1—H12···O22^iii^	0.86 (2)	2.06 (2)	2.897 (8)	164 (6)
O2—H21···O32^iii^	0.86 (2)	2.04 (3)	2.849 (7)	155 (7)
O2—H22···O21^iv^	0.86 (2)	1.90 (2)	2.755 (7)	174 (8)
O13—H131···O41^i^	0.86 (1)	2.13 (3)	2.898 (6)	149 (4)
O13—H132···O1^v^	0.86 (2)	2.02 (3)	2.867 (6)	165 (7)
O23—H232···O13^vi^	0.86 (2)	2.01 (3)	2.807 (6)	154 (5)
O33—H331···O12^vii^	0.86 (2)	1.93 (2)	2.777 (7)	169 (6)
O33—H332···O43^iii^	0.85 (2)	2.31 (3)	3.106 (6)	154 (6)
O43—H431···O22	0.86 (2)	2.03 (2)	2.879 (6)	170 (7)
O43—H432···O2^viii^	0.86 (1)	2.00 (4)	2.773 (6)	148 (5)
O23—H231···O42^ix^	0.86 (1)	1.86 (2)	2.715 (6)	177 (5)

Symmetry codes: (i) *x*, *y*, *z*−1; (iii) −*x*, *y*+1/2, −*z*+1; (iv) *x*, *y*+1, *z*; (v) *x*+1, *y*, *z*; (vi) −*x*+1, *y*−1/2, −*z*; (vii) −*x*+1, *y*+1/2, −*z*+1; (viii) *x*, *y*−1, *z*; (ix) −*x*+1, *y*−1/2, −*z*+1.
